# A Case of Retention of a Dead Ovum in Utero for Six Months, without Putrefaction

**Published:** 1847-10

**Authors:** George L. Upshur

**Affiliations:** Norfolk, Va.


### MEDICAL EXAMINER,

AND

### RECORD OF MEDICAL SCIENCE.

NEW SERIES.—No. XXXIV.—OCTOBER, 1847.

### ORIGINAL COMMUNICATIONS.

### *A Case of Retention of a Dead Ovum in Utero for six months, without Putrefaction.* By George L. Upshur, M. D.

Mrs. ——, æt. 38, the mother of six children, menstruated the first week in November, 1846. She supposes herself to have conceived immediately afterwards. About the middle of the following January, while walking in the street, she felt suddenly, without pain or other premonition, a fluid discharge from the vagina, which, upon hastening home, she discovered was blood. Fourteen days after this, the breasts became flaccid, and the morning sickness, which had been gradually lessening, entirely ceased.

On the 30th of July, about 10 o'clock at night, she was seized with pains like those of labour, which steadily increased in force and frequency until early next morning, when there was expelled a dead fœtus, three inches long, with the membranes and placenta attached by a cord six inches in length. During the whole time, from the first hemorrhage to the expulsion of the ovum, a period of six and a half months, there was more or less sanguineous discharge from the vagina. This discharge was as free from unpleasant odour as the catamenial fluid usually is, except during three days in the month of April, when it was slightly putrid in its character. She consulted no physician about the matter, as the flow was unaccompanied by pain, and never so profuse as to affect her health seriously, or to prevent her from attending to the ordinary duties of the household.

The correctness of this statement may be implicitly relied on. The lady in question is sensible and intelligent, and noted for the particularity with which she marks the various events connected with her pregnancies. After the morning sickness ceased, and the breasts became flaccid, she believed she had aborted and so she expressed herself to her friends, pointing to those symptoms as evidences of the fact.

I had an opportunity of carefully examining the ovum two hours after its expulsion. There was not the slightest sign of putridity about it, the odour resembling very closely that of fresh beef. The placenta was hypertrophied, three and a half inches in diameter and an inch and a half thick, lobulated, very firm under pressure, and nearly exsanguine.

The cord was not uniform in size, nor invested by the membranes, of which a small proportion only were attached to the placenta. The vessels of the cord seem to have coalesced into one, and were impervious. The fœtus was of a variegated mahogany colour, preternaturally firm to the touch, with features just distinguishable. The fingers and toes were scarcely sufficiently developed to be counted, and there was no ossific deposit in any portion of the body. I have the entire ovum as it was expelled, preserved in alcohol.

It is a universally admitted law in obstetrics, that as soon as the ovum dies, it is thrown off from the uterus as a foreign body; or, if it remain any length of time in that organ, it begins to decompose, and is eliminated in a putrid discharge from the vagina. And it seems, indeed, difficult to imagine, how a dead body can be long exposed to the combined action of heat, air, and moisture, without undergoing the putrefactive process.

In the September number of the *Medico Chir. Rev.* for 1823, p. 308, there is an article entitled "Cas Rares," extracted from the *Dict. des Sci. Med.*, by M. Fournier, in which three cases are given of retention of the fœtus for a very long time after its death. The first is cited from Albosius, where the fœtus was retained *twenty-eight* years, and was petrified. In the second, it was retained in utero *thirty-one* years. The woman died of peripneumonia, and, upon examination of the body, the fœtus was found enveloped in chorion and amnion, which were ossified, as well as the placenta. There was no sign of putrefaction about it, nor did it exhale any unpleasant smell. It was about the size of a nine months' child. In the third case, a fœtus was found in the uterus of a woman who had been pregnant *twenty-three* years. It had neither umbilical cord, placenta, nor membranes, and was almost entirely petrified.

These cases seem to be well authenticated, being recommended as both curious and useful, by Dr. Johnson, the then editor of the *Med. Chir. Rev.* I leave these anomalies to be accounted for by older and more experienced heads than mine.

*Norfolk, Va., September 7th,* 1847.

